# Promoting electrocatalytic CO_2_ reduction to *n*-propanol over ethanol at Cu step sites†

**DOI:** 10.1039/d5sc02562a

**Published:** 2025-06-27

**Authors:** Yuanyuan Xue, Ximeng Lv, Chao Yang, Lu Song, Lijuan Zhang, Gengfeng Zheng

**Affiliations:** a Laboratory of Advanced Materials, State Key Laboratory of Porous Materials for Separation and Conversion, Shanghai Key Laboratory of Molecular Catalysis and Innovative Materials, Fudan University Shanghai 200438 China gfzheng@fudan.edu.cn zhanglijuan@fudan.edu.cn

## Abstract

Obtaining valuable C_3+_ products directly from the electrocatalytic reduction of CO_2_ or CO is an attractive but challenging task, due to the much more complicated reaction pathways and sluggish kinetics of C_3+_ products than their C_1_ and C_2_ counterparts. As different C_3+_ products and competitive C_2_ side-products may share the common rate-determining step (*e.g.* the carbon–carbon coupling), the regulation of subsequent selectivity-determining step(s) is critical for promoting the selectivity of C_3+_ products. Herein, we focused on tuning the selectivity competition between *n*-propanol (*n*-C_3_H_7_OH, an important C_3+_ alcohol) *versus* ethanol (C_2_H_5_OH, a major C_2_ side product), based on the constant potential computations on the Cu surface with different step sites. The critical selectivity-determining steps for the *n*-C_3_H_7_OH and C_2_H_5_OH pathways have been identified, and the impact of Cu step sites on the competitive relation between *n*-C_3_H_7_OH and C_2_H_5_OH has been explored. Moreover, a descriptor related closely to the *n*-propanol selectivity has been developed, showing that controlling the competitive hydrogenation of C_2_ intermediates and C_1_–C_2_ coupling processes is vital to differentiate the selectivity of *n*-propanol from ethanol. This work can inspire the screening and rational design of unconventional electrocatalytic sites for generating more value-added C_3+_ products from the electrocatalytic CO_2_ reduction.

## Abstract 2

We demonstrate a selectivity descriptor to evaluate the competition between the CH_3_CHO* protonation and the coupling of CH_3_CHO* and CO*, which are the selectivity-determining steps for the ethanol pathway *versus* the *n*-propanol pathway, respectively.

## Graphical abstract

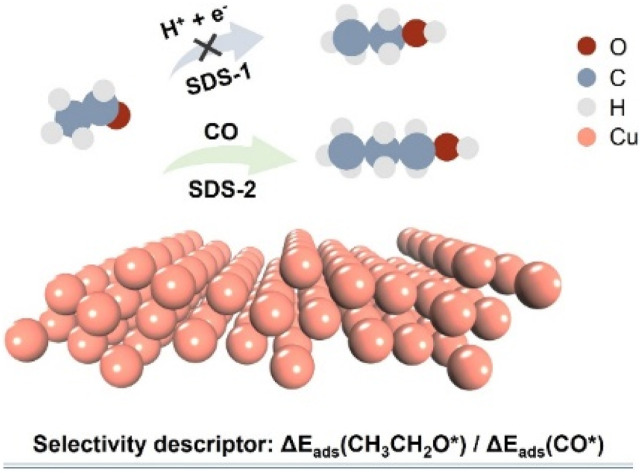

## Introduction

The electrocatalytic CO_2_ or CO reduction reaction (CO_2_RR/CORR) using renewable electricity has attractive potential for reducing carbon footprint and energy storage in liquid fuel products like alcohols,^1–4^ due to their high energy densities, convenient storage, and facile transportation.^5,6^ C_1_ and C_2_ alcohols, *i.e.*, methanol (CH_3_OH)^7–9^ and ethanol (C_2_H_5_OH),^10–12^ have relatively high selectivities and activities. In contrast, the selective electroreduction of CO_(2)_ into C_3+_ alcohols, such as *n*-propanol (*n*-C_3_H_7_OH), is still challenging *versus* the competing side reactions of C_1_ and C_2_ products. As the CO_(2)_-to-C_3_H_7_OH involves complicated reaction pathways containing both the C_1_–C_1_ coupling and subsequent C_1_–C_2_ coupling,^13,14^ most of the reported faradaic efficiencies (FEs) of *n*-C_3_H_7_OH in CO_(2)_ electroreduction are still below 20% to date.^15–18^

A variety of approaches have been investigated to promote the selectivity of the *n*-propanol product from the CO_(2)_RR. For instance, doping Au into Cu(100) was reported to decrease the adsorption of CO* (where * refers to the adsorption site) while retaining the intrinsic Cu(100) active sites at the same time, which facilitated the C_1_–C_2_ and C_1_–C_1_ coupling process and presented a peak FE of 18% for *n*-C_3_H_7_OH.^15^ Cu co-doped with Ag and Ru was synthesized for the CO electroreduction to *n*-C_3_H_7_OH, with a 37% FE and >100 mA cm^−2^ of partial current density.^19^ Nonetheless, the production selectivity and yield of *n*-C_3_H_7_OH by the electrocatalytic CO_(2)_RR are still much lower than those of the C_1_ and C_2_ side products and also far from the commercialization requirements.^20–22^

The selectivity of C_1_ and C_2_ products in the CO_(2)_RR can be promoted based on the rate-determining step (RDS) regulation,^23–29^ such as using atomic structure design^23^ or microenvironmental tuning.^24,26^ However, as the C_3_ formation steps (*e.g.* the C_1_–C_2_ coupling and the hydrogenation of C_3_ intermediates) are far away from the initial reaction stage and unlikely to serve as the RDS,^30^ different C_3+_ products and those C_2_ side products may share the same RDS. Thus, it is hard to improve the selectivity of C_3+_ products by the RDS tuning strategy. The selectivities of C_3+_ products should mainly be determined by the selectivity-determining steps (SDSs) for the competitive pathways.^31^ Ethanol has been proposed as a major competing side product of *n*-C_3_H_7_OH.^32–36^ Wang and coworkers analyzed the reported CO_2_RR-relevant studies using the machine learning method and found correlation between FEs/ΔFEs of ethanol and *n*-propanol, suggesting that ethanol and *n*-propanol share the common C–C coupling process and compete with each other.^36^ In addition, according to our previously reported work,^2^ the pathways to ethanol and *n*-propanol separate with the acetate/acetic acid pathway at an earlier stage (CH_2_CO*). Thus, the FE of acetate is generally low under conditions that are advantageous for the *n*-propanol formation.^37,38^ Thus, the competitive relationship between ethanol and *n*-propanol is more critical for determining the *n*-propanol selectivity in the CO_(2)_RR. By using differential electrochemical mass spectrometry, it was found that the concentration ratios of acetaldehyde/ethanol and propionaldehyde/*n*-propanol near the cathode surface are higher than those in the bulk electrolyte during CO_2_ electroreduction, suggesting that acetaldehyde (CH_3_CHO) is the bifurcation point of C_2_H_5_OH and *n*-C_3_H_7_OH.^32^ The subsequent coupling of CH_3_CHO and CO* can lead to the formation of *n*-C_3_H_7_OH, while the further hydrogenation of CH_3_CHO results in C_2_H_5_OH.^32^ In addition to acetaldehyde, methylcarbonyl (CH_3_CO*) has also been suggested as another possible branching point for the C_2_H_5_OH and *n*-C_3_H_7_OH pathways.^34^ Nonetheless, despite that they are crucial for the CO_(2)_RR to *n*-C_3_H_7_OH, the branching intermediates and selectivity-determining steps for the C_2_H_5_OH and *n*-C_3_H_7_OH pathways are still ambiguous, precluding the breakthrough of designing efficient electrocatalysts.

In this work, we first conducted constant potential computations to identify the selectivity-determining steps and the critical bifurcation intermediate for the *n*-C_3_H_7_OH and C_2_H_5_OH pathways. Then we designed a variety of high-index Cu facets with step sites and theoretically investigated for their catalytic performances on the selectivity competition between the *n*-C_3_H_7_OH and C_2_H_5_OH pathways. Finally, a critical descriptor was developed to predict the capabilities of different Cu sites for the CO_(2)_RR to *n*-C_3_H_7_OH, suggesting the potential of developing new electrocatalysts for more value-added products.

## Results and discussion

### Selectivity mechanism

**Fig. 1:**
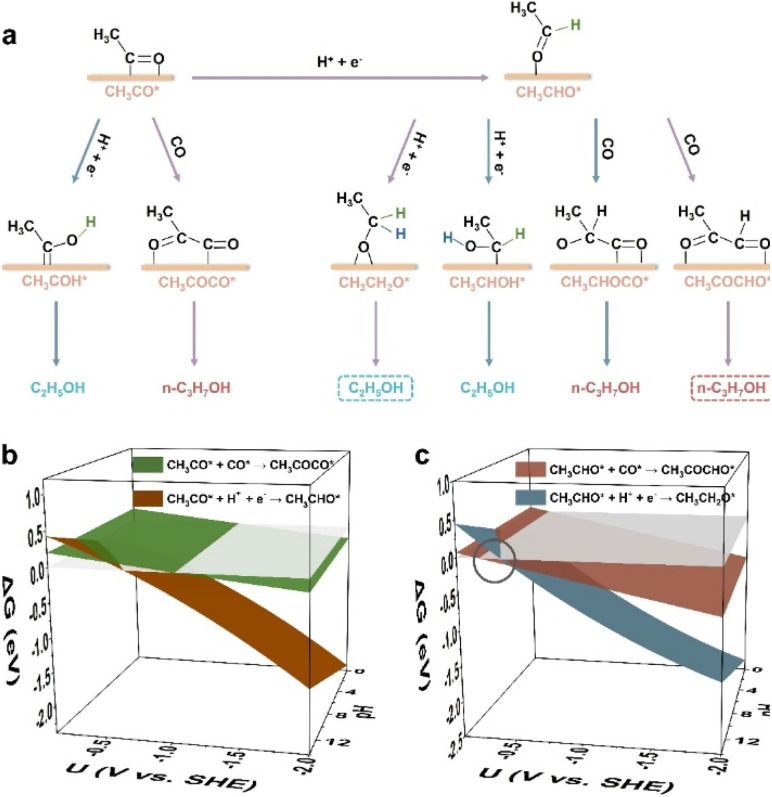
(a) Possible hydrogenation and coupling steps of the two possible branching intermediates (CH_3_CO* and CH_3_CHO*) for C_2_H_5_OH and *n*-C_3_H_7_OH pathways. The preferable hydrogenation and coupling steps of CH_3_CO* and CH_3_CHO* are marked with purple arrows. The hydrogen atoms from the hydrogenation of CH_3_CO* are shown in green color, and the hydrogen atoms from the hydrogenation of CH_3_CHO* are shown in blue color. The most possible C_2_H_5_OH and *n*-C_3_H_7_OH pathways are highlighted with the dashed boxes. (b) Free energy changes of the hydrogenation and coupling steps of CH_3_CO* on Cu(100) *versus* the potential and pH. (c) Free energy changes of the hydrogenation and coupling steps of CH_3_CHO* on Cu(100) *versus* the potential and pH. The circle highlights the dominant potential range (at pH 14) where the coupling step proceeds preferably. The grey planes in (b) and (c) are the planes with the function of Δ*G* = 0 (eV).

As Cu(100) has been widely reported for the CO_(2)_RR to C_2+_ products (mostly C_2_ products like ethylene and ethanol though),^39^ we first conducted constant potential calculations to explore the critical elementary steps regarding the competition between C_2_H_5_OH and *n*-C_3_H_7_OH pathways on Cu(100) (computational details in Fig. S1 and Tables S1, S2†). There are two possible bifurcation intermediates (*i.e.*, CH_3_CO* and CH_3_CHO*) for the competition pathways between C_2_H_5_OH and *n*-C_3_H_7_OH,^32–34^ and the possible hydrogenation steps and coupling steps of those two intermediates are schematically displayed (Fig. 1a). Although CH_2_CHO* has also been proposed as a possible precursor to form CH_3_CHO*,^37^ the formation of CH_3_CO is easier than that of CH_2_CHO* (Fig. S2†). Thus, CH_3_CO* is chosen as the starting point (Fig. 1a). For the hydrogenation of CH_3_CO*, the free energy change (Δ*G*) to CH_3_CHO* (*i.e.*, CH_3_CO* + H^+^ + e^−^ → CH_3_CHO*) is more negative than that of CH_3_COH* (*i.e.*, CH_3_CO* + H^+^ + e^−^ → CH_3_COH*) in the whole potential range and pH range (Fig. S3†), indicating that the carbon atom of the carbonyl group in CH_3_CO* tends to obtain the proton rather than the oxygen atom of the carbonyl group. For the subsequent hydrogenation of CH_3_CHO*, the carbon atom of the aldehyde group is also easier to obtain the proton (*i.e.*, CH_3_CH_2_O*) than the oxygen atom of the aldehyde group (*i.e.*, CH_3_CHOH*) (Fig. S4†), suggesting that CH_3_CH_2_O* is more likely to be the key intermediate toward ethanol than CH_3_CHOH*. For the *n*-propanol formation pathway (Fig. S5†), the coupling of CH_3_CHO* with CO* tends to form CH_3_COCHO* on Cu(100) within the whole potential and pH ranges, rather than form the CH_3_CHOCO* intermediate.

From the above analysis, the most possible hydrogenation and coupling steps of CH_3_CO* and CH_3_CHO* are determined (purple arrows in Fig. 1a), among which CH_3_CHO* can be obtained from the hydrogenation of CH_3_CO*. As shown in Fig. 1b, the coupling between CH_3_CO* and CO* (*i.e.*, CH_3_CO* + CO* → CH_3_COCO*) is preferable under alkaline conditions, as the Δ*G* of the CH_3_CO* hydrogenation step (*i.e.*, CH_3_CO* + H^+^ + e^−^ → CH_3_CHO*) is more positive in a higher pH environment. However, when the coupling step becomes spontaneous, Δ*G* of the CH_3_CO* protonation step is more negative, even at pH 14. Thus, the protonation of CH_3_CO* to CH_3_CHO* is generally advantageous during the CO_(2)_RR. On the other hand, for CH_3_CHO* in an alkaline environment (Fig. 1c), the coupling step (CH_3_CHO* + CO* → CH_3_COCHO*) is more preferable than its protonation step in the potential range of −0.27 to −0.50 V *vs.* the standard hydrogen electrode (SHE) at pH 14, suggesting that CH_3_CHO* is more likely to be the branching intermediate for C_2_H_5_OH and *n*-C_3_H_7_OH pathways. The corresponding SDS for C_2_H_5_OH formation is: CH_3_CHO* + H^+^ + e^−^ → CH_3_CH_2_O*, and the corresponding SDS for *n*-C_3_H_7_OH formation is: CH_3_CHO* + CO* → CH_3_COCHO*. Kastlunger *et al.* conducted microkinetic simulations based on the constant-potential density functional theory (DFT) to explore the formation of C_2_ products by the CO_2_RR on Cu(100)^40^ and found that the hydrogenation of CH_3_CHO* to CH_3_CH_2_O* led to the formation of C_2_H_5_OH, consistent with our results. Recently, the surface reconstruction of Cu(100) during the CO_2_RR was theoretically explored by the potential-dependent grand canonical Monte Carlo method combined with the environmental kinetic Monte Carlo method and the DFT method, showing that C_2_H_5_OH can be produced through the hydrogenation of CH_3_CHO* to CH_3_CH_2_O*.^41^ This work also supports that the hydrogenation of CH_3_CHO* is a critical step for the formation of C_2_H_5_OH. The free energy profiles of SDSs for both the C_2_H_5_OH and *n*-C_3_H_7_OH formation pathways at −0.4 V *vs.* SHE at pH 14 are displayed (Fig. S6†), indicating the feasibility for the CO_(2)_RR to *n*-propanol *via* the coupling between CH_3_CHO* and CO*.

When the potential becomes more negative (<−0.50 V *vs.* SHE, pH 14), the hydrogenation step of *CH_3_CHO toward ethanol becomes more dominant than the coupling step on Cu(100) (Fig. 1c), indicating that the perfect Cu(100) facet is hard to catalyze the CO_(2)_RR to *n*-C_3_H_7_OH. In comparison, on Ag-doped Cu, the SDS for the *n*-C_3_H_7_OH pathway becomes dominant in the potential range between 0.22 and −0.96 V *vs.* SHE at pH 14 (see Δ*G*(U, pH) and structures in Fig. S7, computational details in Fig. S8 and Table S3†), in accordance with the experimental observation of the enhanced *n*-C_3_H_7_OH selectivity on Ag-doped Cu,^42^ also confirming the branching intermediate (CH_3_CHO*) and SDSs for C_2_H_5_OH and *n*-C_3_H_7_OH pathways.

### Step effects

**Fig. 2:**
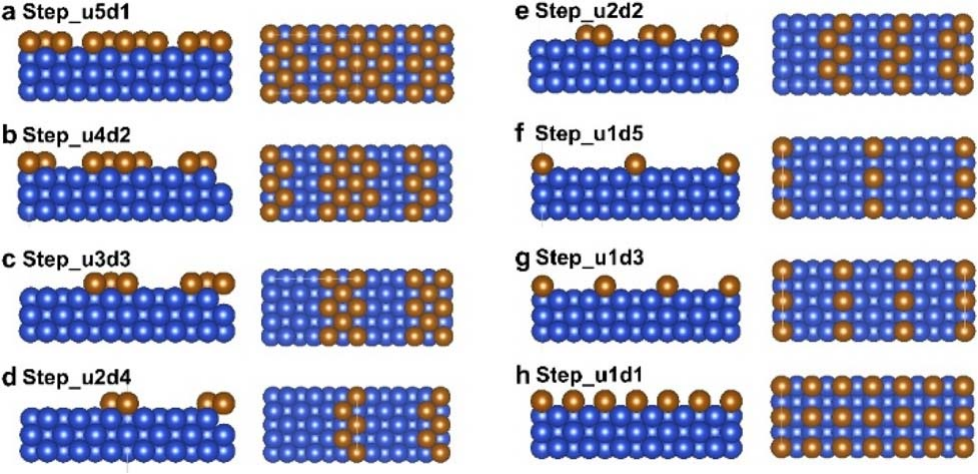
(a–h) The side views and top views of different step surfaces constructed based on the Cu(100) facet, including (a) Step_u5d1, (b) Step_u4d2, (c) Step_u3d3, (d) Step_u2d4, (e) Step_u2d2, (f) Step_u1d5, (g) Step_u1d3, and (h) Step_u1d1. The Cu atoms of the uppermost layer are presented with a brown color to clearly display the step sites. These step surfaces were denoted as “Step_u(*x*)d(*y*)”, which means that the upper terrace width of the step unit is “*x*” times the diameter of the Cu atom, and the lower terrace width of the step unit is “*y*” times the diameter of the Cu atom. The diameter of the Cu atom is 1.8 Å.

**Fig. 3:**
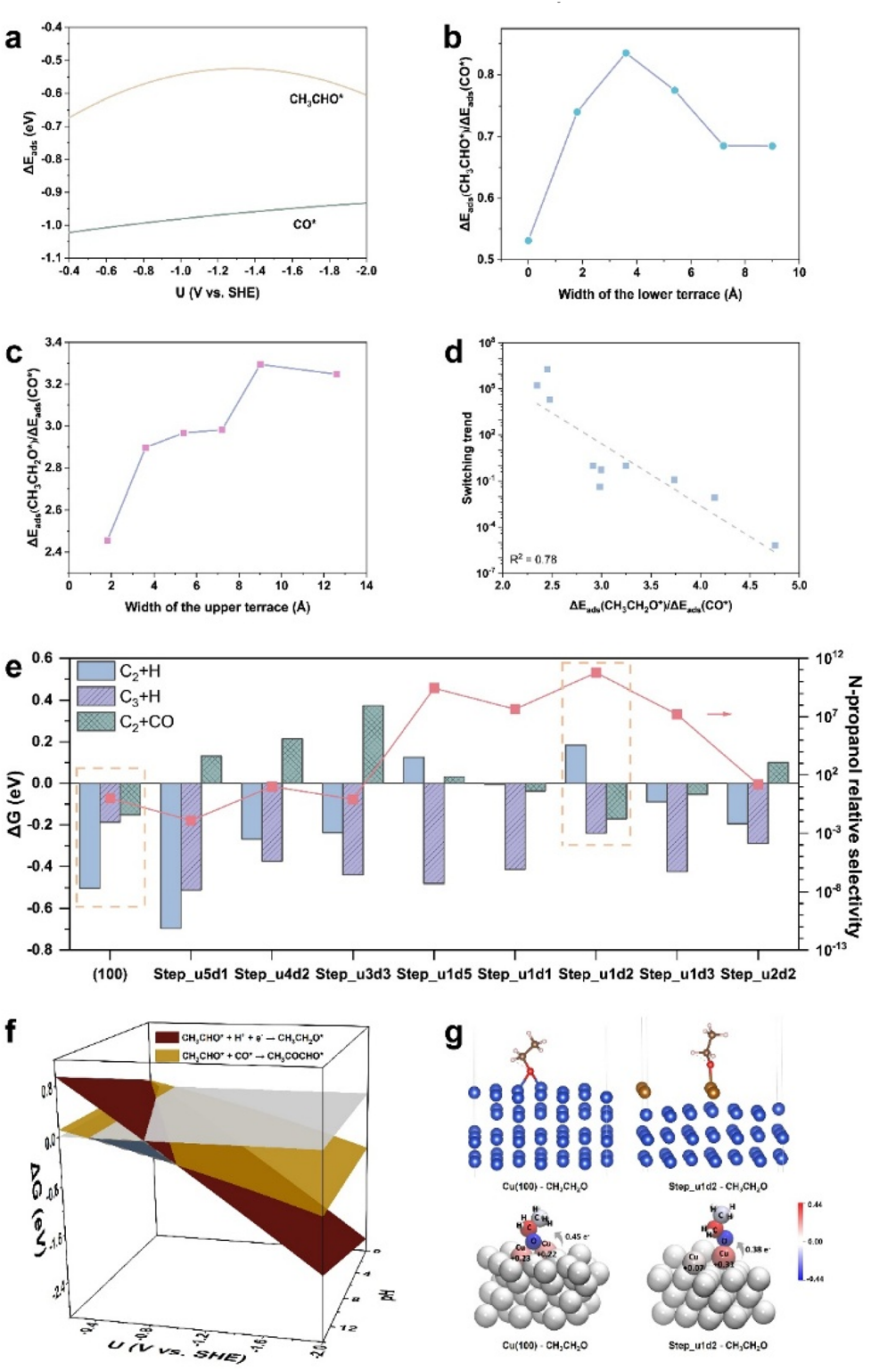
(a) The adsorption energies (Δ*E*_ads_) of CH_3_CHO* and CO* on Cu(100) against the potential. (b) The adsorption energy ratios between CH_3_CHO* and CO* of the step surfaces constructed based on Cu(100) against the width of the lower terrace. (c) The adsorption energy ratios between CH_3_CH_2_O* and CO* of the step surfaces constructed based on Cu(100) against the width of the upper terrace. The data in (b) and (c) are from Cu(100), Step_u5d1, Step_u4d2, Step_u3d3, Step_u2d4, and Step_u1d5. (d) The relation of the switching trend (defined as *K*_C_2_+CO_/*K*_C_2_+H_) against the descriptor Δ*E*_ads_(CH_3_CH_2_O*)/Δ*E*_ads_(CO*). (e) The free energy changes of three reaction steps including the protonation of CH_3_CHO* (C_2_ + H), the protonation of CH_3_COCHO* (C_3_ + H), and the coupling between CH_3_CHO* and CO* (C_2_ + CO), and *n*-propanol relative selectivity of Cu(100) and step surfaces constructed based on Cu(100). (f) The free energy changes of the SDSs for *n*-propanol and ethanol pathways on Step_u1d2 against the potential and pH. The grey plane is the plane with the function of Δ*G* = 0 (eV). The highlighted region with blue color shows the potential range at pH = 14 where the *n*-propanol is preferably produced. (g) The adsorption configurations of CH_3_CH_2_O* on Cu(100) and Step_u1d2 (top), and the atomic charge coloring diagrams of CH_3_CH_2_O* on Cu(100) and Step_u1d2 (bottom), the numbers of electron transferred from the surface adsorption sites to CH_3_CH_2_O* are marked.

After determining the critical branching intermediate and corresponding SDSs for the C_2_H_5_OH and *n*-C_3_H_7_OH pathways, we further investigated the roles of surface step sites in the competition between C_2_H_5_OH and *n*-C_3_H_7_OH. The explicit functions of step sites on the *n*-C_3_H_7_OH selectivity were first surveyed by constructing surface steps with different upper terrace widths and lower terrace widths based on the Cu(100) facet (Fig. 2a–h). The step surfaces were constructed by removing the different numbers of atom row on the top layer of Cu(100), and the width of one row is the diameter of Cu (1.8 Å). The formed step surfaces are designated as “Step_u(*x*)d(*y*)”, where “u(*x*)d(*y*)” refers to the step site unit comprising *x* rows at the upper terrace and *y* rows at the lower terrace. It was found that the adsorption of CH_3_CHO* and CO* competes with each other,^33^ while the adsorption of CH_3_CHO* on Cu(100) is always weaker than that of CO* in the whole potential range of the CO_(2)_RR (Fig. 3a, computational details in Fig. S9 and Table S4†). For the coupling of CH_3_CHO* and CO* (*i.e.*, the SDS for the *n*-propanol pathway), the adsorption of both CO* and CH_3_CHO* should be optimized. Thus, the Δ*E*_ads_(CH_3_CHO*)/Δ*E*_ads_(CO*) ratio is used to evaluate the priority of the *n*-C_3_H_7_OH pathway, from which the ratio close to 1 suggests an optimal match of both CH_3_CHO* and CO* adsorption. The Δ*E*_ads_(CH_3_CHO*)/Δ*E*_ads_(CO*) ratio reaches the highest value of 0.84 when the width of the lower terrace of the Cu(100) step is 3.6 Å (Fig. 3b, computational details in Table S5†). On the other hand, for the protonation of CH_3_CHO* to CH_3_CH_2_O* (*i.e.*, the SDS of the C_2_H_5_OH pathway), when hydrogenated CH_3_CH_2_O* is more stable, the possibility for the formation of C_2_H_5_OH increases. Thus, the Δ*E*_ads_(CH_3_CH_2_O*)/Δ*E*_ads_(CO*) ratio is used to represent the protonation capability of the catalyst for C_2+_ intermediates, from which the smaller ratio represents that the hydrogenation step is less likely to occur. The Δ*E*_ads_(CH_3_CH_2_O*)/Δ*E*_ads_(CO*) ratio reaches the lowest value (2.45) when the width of the upper terrace is 1.8 Å (Fig. 3c, computational details in Table S5†). Based on the two indicators above, the optimal Cu(100) step is the Step_u1d2, with a lower terrace width of 3.6 Å and an upper terrace width of 1.8 Å (Fig. S10†).

To evaluate the *n*-C_3_H_7_OH selectivity of different step sites, we set the Cu(100) surface as the benchmark, and the *n*-C_3_H_7_OH relative selectivity compared to the Cu(100) surface is defined as: (*K*_C_2_+CO_/*K*_C_2_+H_ × *K*_C_3_+H_), where *K* = *k*_step_/*k*_Cu(100)_, *k* refers to the rate constant of an elementary reaction, “step” refers to the step surfaces, and “C_2_ + CO”, “C_2_ + H”, and “C_3_ + H” represent the coupling of CH_3_CHO* and CO* to CH_3_COCHO*, the hydrogenation of CH_3_CHO* to CH_3_CH_2_O*, and the hydrogenation of CH_3_COCHO* to CH_3_COCHOH* (Fig. S11†), respectively. The relative *n*-C_3_H_7_OH selectivity of the Cu(100) surface is set as 1. “*K*_C_2_+CO_/*K*_C_2_+H_” represents the switching trend of the C_2_H_5_OH and *n*-C_3_H_7_OH pathways, which shows a linear correlation with the Δ*E*_ads_(CH_3_CH_2_O*)/Δ*E*_ads_(CO*) value (Fig. 3d). Then the relative selectivity of *n*-C_3_H_7_OH on different Cu(100) steps was calculated, among which the Step_u1d2 sites show the highest *n*-C_3_H_7_OH relative selectivity of 5.7 × 10^10^ (Fig. 3e, right *y*-axis). The SDS of the *n*-C_3_H_7_OH pathway on Step_u1d2 is more dominant than the ethanol pathway in the potential range of −0.41 to −1.03 V *vs.* SHE at pH 14 (Fig. 3f, computational details in Fig. S12 and Table S6†), wider than that of the perfect Cu(100) surface (Fig. 1c, −0.27 to −0.50 V *vs.* SHE). By comparing Δ*G* values of the hydrogenation and coupling steps of CH_3_CHO* and the hydrogenation step of CH_3_COCHO* on Step_u1d2 and Cu(100) (Fig. 3e, left *y*-axis), the suppression of the CH_3_CHO* protonation contributes the most to the enhanced *n*-C_3_H_7_OH relative selectivity of Step_u1d2. The adsorption of CH_3_CH_2_O* is switched from a bridged-adsorption mode on the Cu(100) surface, to a top-adsorption mode on the Step_u1d2 sites due to the confined surface structure (Fig. 3g). This top-adsorption mode leads to the less electron transfer from Cu atoms to CH_3_CH_2_O* according to the Bader charge and differential charge density analysis (Fig. 3g and S13†), thus decreasing the binding strength of CH_3_CH_2_O* on Step_u1d2 (Fig. S14a, computational details in Fig. S12 and Table S6†). On the other hand, the adsorption of CH_3_CHO* on Step_u1d2 is stronger than that on Cu(100) (Fig. S14b, computational details in Fig. S12 and Table S6†). The angle between the Cu–O bond (the O atom from CH_3_CHO*) and the surface plane of Step_u1d2 is 64° (Fig. S15†), smaller than that of CH_3_CHO* on Cu(100) (82°), indicating a geometric affinity of Step_u1d2 for the CH_3_CHO* adsorption. Thus, the weak adsorption of CH_3_CH_2_O* and the strong adsorption of CH_3_CHO* on Step_u1d2 together contribute to the inhibited protonation of CH_3_CHO* and enhanced *n*-C_3_H_7_OH relative selectivity.

**Fig. 4:**
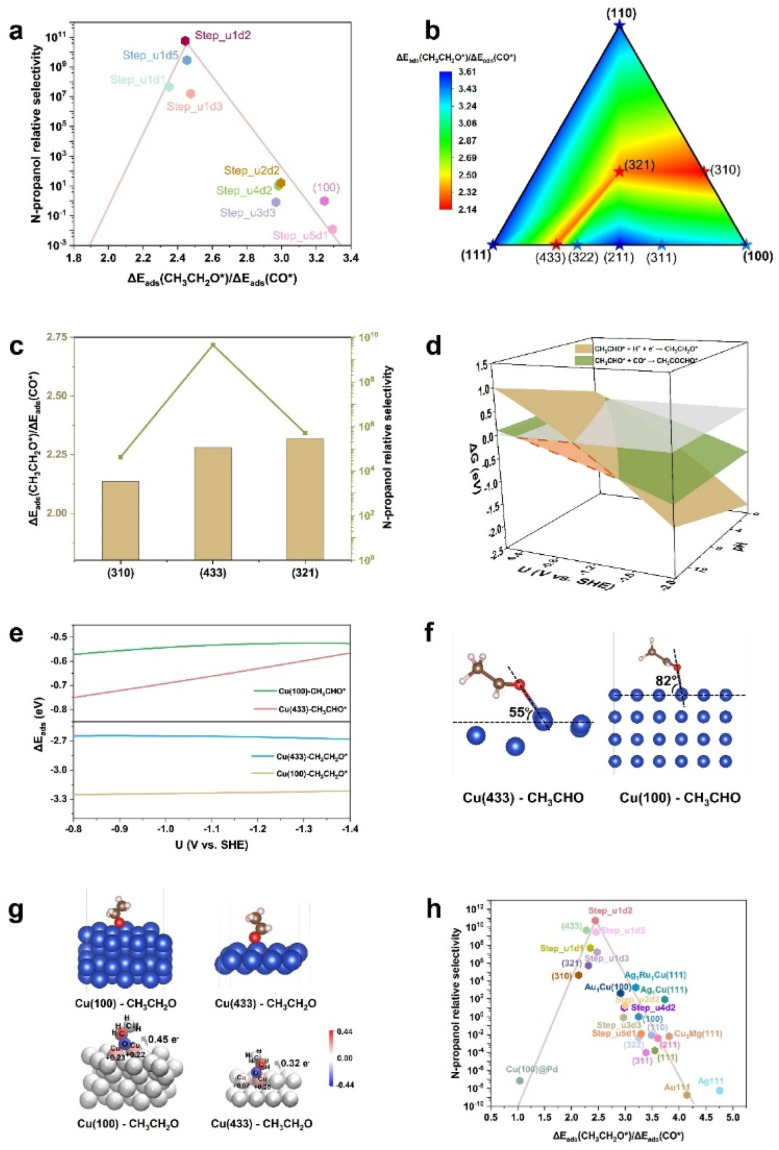
(a) The volcano plot of *n*-propanol relative selectivity (defined as *K*_C_2_+CO_/*K*_C_2_+H_ × *K*_C_3_+H_) *versus* the descriptor Δ*E*_ads_(CH_3_CH_2_O*)/Δ*E*_ads_(CO*). (b) The contour map showing the Δ*E*_ads_(CH_3_CH_2_O*)/Δ*E*_ads_(CO*) values of different Cu facets. (c) The *n*-propanol relative selectivity and Δ*E*_ads_(CH_3_CH_2_O*)/Δ*E*_ads_(CO*) values of three efficient Cu facets for the CO_(2)_RR to *n*-propanol. (d) The free energy changes of the SDSs for *n*-propanol and ethanol pathways on Cu (433) against the potential and pH. The grey plane is the plane with the function of Δ*G* = 0 (eV). The highlighted region with orange color shows the potential range at pH = 14 where the *n*-propanol is preferably produced. (e) The adsorption energies of CH_3_CHO* (top) and CH_3_CH_2_O* on Cu(100) and Cu(433) against the potential. The potential range from −0.8 to −1.4 V *vs.* SHE is where the formation of *n*-propanol is preferable on Cu(433). (f) The adsorption configurations of CH_3_CHO* on Cu(433) and Cu(100). The angles between the Cu–O bond and the surface are marked. (g) The adsorption configurations of CH_3_CH_2_O* on Cu(100) and Cu(433) (top) and the atomic charge coloring diagrams of CH_3_CH_2_O* on Cu(100) and Cu(433) (bottom), the numbers of electron transferred from the surface adsorption sites to CH_3_CH_2_O* are marked. (h) The volcano plot of the *n*-propanol relative selectivity *versus* the descriptor Δ*E*_ads_(CH_3_CH_2_O*)/Δ*E*_ads_(CO*), including the data of the step surfaces (the Step_u(*x*)d(*y*) surfaces and Cu facets), the Cu-based bimetals, and other metals.

Furthermore, the *n*-C_3_H_7_OH relative selectivity of Cu(100) and step sites shows a volcano trend with the Δ*E*_ads_(CH_3_CH_2_O*)/Δ*E*_ads_(CO*) value (Fig. 4a), as the adsorption energies of different reaction intermediates are correlated during the reactions.^43^ When Δ*E*_ads_(CH_3_CH_2_O*)/Δ*E*_ads_(CO*) decreases at the right side of the volcano, the hydrogenation step of CH_3_CHO* (*i.e.*, SDS for the C_2_H_5_OH pathway) is inhibited as the adsorbed CH_3_CH_2_O* becomes unstable. This SDS suppression of the C_2_H_5_OH pathway is beneficial for the *n*-C_3_H_7_OH production. When Δ*E*_ads_(CH_3_CH_2_O*)/Δ*E*_ads_(CO*) further decreases at the left side of the volcano, not only the protonation of CH_3_CHO* is suppressed, but also the protonation of C_3_ intermediates, like CH_3_COCHO*, is also suppressed. Thus, the *n*-C_3_H_7_OH relative selectivity decreases as the Δ*E*_ads_(CH_3_CH_2_O*)/Δ*E*_ads_(CO*) further decreases (at the left side of the volcano).

### Facet prediction

As the high-index facets of Cu show characteristics of different step sites, we further screened the potential facets for the electroreduction of CO_(2)_ toward *n*-C_3_H_7_OH using the Δ*E*_ads_(CH_3_CH_2_O*)/Δ*E*_ads_(CO*) descriptor (Fig. 4b). The Δ*E*_ads_(CH_3_CH_2_O*)/Δ*E*_ads_(CO*) values of (433), (321), and (310) are located in the optimal range (2.0−3.0 eV). In our work, the high-index facets have been constructed from the primitive cell of Cu, to control the suitable model size for DFT computations. For instance, Cu(321) studied in this work corresponds to Cu(210) (Fig. S16†), and a distinct experiment performance of the Cu(210) facets for the CO_2_RR to *n*-propanol was previously reported,^44^ further confirming the practicability of the selectivity descriptor.

Compared to different facets, Cu(433) exhibits the highest relative selectivity (∼10^9^) of *n*-propanol (Fig. 4c). The potential range for *n*-propanol production on Cu(433) was calculated to be −0.40 to −1.49 V *vs.* SHE at pH 14 (Fig. 4d, computational details in Fig. S17 and Table S7†), which covers the experimentally observed potential range (−1.20 to −1.50 V *vs.* SHE, at pH 14) for *n*-propanol production,^15,20^ further indicating the great potential of Cu(433) in the CO_(2)_RR to *n*-propanol. Compared to Cu(100), Cu(433) shows a stronger adsorption for CH_3_CHO* and a weaker adsorption for CH_3_CH_2_O* in the potential range for *n*-propanol production (Fig. 4e, computational details in Fig. S17 and Table S7†). Thus, the hydrogenation of CH_3_CHO* on Cu(433) becomes difficult and the ethanol pathway is inhibited. The strong adsorption of CH_3_CHO* on Cu(433) is attributed to the geometric effect from the step sites. Compared to Cu(100), CH_3_CHO* adsorbed on Cu(433) is closer to the surface (Fig. 4f), allowing a strong interaction between the CH_3_CHO* and the Cu(433) surface. On the other hand, CH_3_CH_2_O* is adsorbed at the bridged-sites on Cu(100), and at the top-sites on Cu(433) (Fig. 4g). The less electron transfer from Cu(433) to the adsorbed CH_3_CH_2_O* results in the weak adsorption of CH_3_CH_2_O* based on the Bader charge and differential charge density analysis (Fig. 4g and S18†).

To more clearly show the practicability of the selectivity descriptor Δ*E*_ads_(CH_3_CH_2_O*)/Δ*E*_ads_(CO*), the experimentally reported Cu(321) facet was compared with the Cu(100) and Cu(433) facets. As shown in Fig. S19,† the high *n*-propanol relative selectivity of Cu(321) is also mainly from its capability for inhibiting the hydrogenation of CH_3_CHO*. The binding strength of Cu(321) for CH_3_CHO* is stronger than that of Cu(100) and weaker than that of Cu(433) (Fig. S20†). The adsorption configuration of CH_3_CHO* adsorbed on Cu(321) was analyzed (Fig. S21†). The angle between the Cu–O bond and the surface plane is smaller than that of Cu(100) (82°) and larger than that of Cu(433) (55°), suggesting that the capability of Cu(321) to stabilize the CH_3_CHO* intermediate is superior to that of Cu(100) and inferior to that of Cu(433). On the other hand, the adsorption of CH_3_CH_2_O* on Cu(321) is weaker than that on Cu(100) and stronger than that on Cu(433) (Fig. S22†). Furthermore, CH_3_CH_2_O* is also adsorbed on Cu(321) in a top-adsorption way, and the charge transfer of Cu(321) to the CH_3_CH_2_O* intermediate is less than that of Cu(100) and more than that of Cu(433) (Fig. S23†), confirming that the capability of Cu(321) to adsorb CH_3_CH_2_O* is between that of Cu(100) and Cu(433). Therefore, the *n*-propanol relative selectivity of Cu(321) is higher than that of Cu(100) and lower than that of Cu(433) (Fig. 4c). On Cu(321), the preferable potential range (at pH 14) for the coupling of CH_3_CHO* with CO* is 0 to −0.75 V *vs.* SHE according to the constant potential calculations (Fig. S24, computational details in Fig. S25, and Table S8†). The overall selectivities of Cu(100), Cu(321), and Cu(433) for *n*-propanol were further calculated by considering the mainly competitive carbon-containing products (methane, methanol, ethylene, and ethanol) in the CO_(2)_RR to *n*-propanol (Fig. S26†). Cu(100) was also used as a reference in those calculations. The *n*-propanol overall selectivities (by considering all the possible carbon-containing products) on Cu(433) and Cu(321) are calculated to be ∼10^9^ and ∼10^6^, respectively (Fig. S27†), which are close to the *n*-propanol relative selectivities of the two facets (Fig. 4c). This result confirms that the *n*-propanol relative selectivity is a reasonable metric to evaluate the *n*-propanol selectivity of different structures.

Finally, the relative selectivities of *n*-propanol of all step surfaces (including the step surfaces based on Cu(100) and different Cu facets), the Cu-based bimetals (structures in Fig. S28†), and other metals (structures in Fig. S29†), with respect to the descriptor Δ*E*_ads_(CH_3_CH_2_O*)/Δ*E*_ads_(CO*), exhibit a volcano correlation (Fig. 4h). This result suggests that the selectivity descriptor Δ*E*_ads_(CH_3_CH_2_O*)/Δ*E*_ads_(CO*) is universal in finding the various catalysts for the CO_(2)_RR to *n*-propanol. The Step_u1d2 sites and Cu(433) are located at the top of the volcano plot, suggesting that the capability of those surface Cu catalytic sites toward higher CO_(2)_-to-*n*-propanol conversion selectivities. Although the step surfaces may experience reconstruction during the CO_(2)_RR due to the high surface energies and the harsh reaction conditions, there have been some reports those have successfully synthesized the high-index Cu-based facets and retained good reaction stability.^44–46^ For example, by utilizing OH^−^ anions as the controlling reagents and the ascorbic acid for the slow growth of the nanocrystals, the Cu_2_O(211) facets were synthesized, showing a FE_C_2_H_4__ of 87% in the CO_2_RR after being stored in 1 M KOH for one month.^45^ In addition, it has been found that the presence of the low-index facets can help to stabilize the high-index facets under electroreduction conditions.^46^ Those studies can inspire the synthesis of high-index Cu-based facets for the CO_(2)_RR catalysis.

## Conclusions

In summary, this work represents a rational theoretical design for the electrocatalytic sites for efficient CO_(2)_-to-C_3+_ products based on the constant potential computations. For the formation of *n*-propanol, ethanol shares the common RDS and is a main side product. In our work, CH_3_CHO* has been identified as the critical intermediate for the bifurcation of *n*-propanol and ethanol pathways, and Δ*E*_ads_(CH_3_CH_2_O*)/Δ*E*_ads_(CO*) has been proposed as a key descriptor for the formation of *n*-propanol. Based on this descriptor, different step sites have been screened to select the optimal catalytic sites, and Cu(433) facets have been suggested as the most promising facets for the electrochemical CO_(2)_-to-*n*-propanol conversion. Our work highlights the significance of SDS regulation in the CO_(2)_RR and allows understanding the competition mechanism between the C_2_ and C_3+_ products.

## Author contributions

Y. Xue and G. Zheng designed the research. L. Zhang and G. Zheng supervised the research. Y. Xue, X. Lv, C. Yang, and L. Song performed the research and analyzed the data. Y. Xue and G. Zheng wrote the manuscript. All the authors discussed, commented on and revised the manuscript.

## Conflicts of interest

There are no conflicts to declare.

## Supplementary Material

SC-016-D5SC02562A-s001

## Data Availability

All data supporting this work are included in the ESI.†
