# Supplementary material for: Promoting electrocatalytic CO2 reduction to n-propanol over ethanol at Cu step sites
Source: Chem Sci. 2025 Jun 27;16(30):13944–50. doi: 10.1039/d5sc02562a (PMC12230853; doi:10.1039/d5sc02562a)
Supplement: SC-016-D5SC02562A-s001 [file SC-016-D5SC02562A-s001.pdf]

Supplementary Information (SI) for Chemical Science.
This journal is © The Royal Society of Chemistry 2025

Electronic supplementary information (ESI)

Promoting Electrocatalytic CO_2 Reduction to n-Propanol over Ethanol at Cu Step Sites

Yuanyuan Xue, Ximeng Lv, Chao Yang, Lu Song, Lijuan Zhang* and Gengfeng Zheng*

Laboratory of Advanced Materials, State Key Laboratory of Porous Materials for Separation and Conversion, Shanghai Key Laboratory of Molecular Catalysis and Innovative Materials, Fudan University, Shanghai, 200438, China.

**Email: gfzheng@fudan.edu.cn (G.Z), zhanglijuan@fudan.edu.cn (L.Z.)*

Computational Methods

Modeling

DFT computations are carried out using the Vienna Ab initio Simulation Package (VASP).[1] The Perdew-Burke-Enrzerhof (PBE) parametrization of the generalized gradient approximation (GGA) is used to describe the electronic exchange-correlation energies.[2] And the dispersion correction (D3-Grimme) is employed to better describe the weak interactions.[3] The Cu primitive cell with a face-center structure is first optimized under an energy cutoff of 600 eV. Then the Cu crystal planes, Ag-doped Cu, and step surfaces constructed based on the Cu(100) facet, are modeled to explore the selectivity mechanism of $CO_{(2)}RR$ to n-propanol and step effects. All slabs include four layers, the bottom two layers are fixed to reflect the bulk structure of Cu and the top two layers can relax. During the structure relaxation, the setup of convergence criterion for residual force and energy are 0.03 eV/Å and 10^{-5} eV, respectively. An energy cutoff of 400 eV for the plane-wave basis set is used for all computations except the optimization of the Cu primitive cell. The Ag-doped Cu is constructed by placing a copper atom on the top layer of Cu(100) with a silver atom. The Cu(100) step surfaces were constructed by removing the different numbers of atom row on the top layer of Cu(100). The width of one row was the diameter of Cu (1.8 Å). The formed step surfaces were named as “Step_u(x)d(y)”, where “u(x)d(y)” refers to the step site unit comprising x rows at the upper terrace and y rows at the lower terrace. The high-index planes of Cu are constructed from the optimized Cu primitive cell for controlling the suitable size of the simulation cell. A vacuum space of more than 10 Å is employed along the z-axis to avoid the

interaction between two periodic units. The coverages of different intermediates keep close on different stabs by controlling the size of the simulation cell or the number of intermediates adsorbed on the stab. Brillouin zones are sampled by a Gamma-centered Monkhorst-Pack k-point mesh with 4 × 1 × 1 for Step_u1d3 and Step_u2d2. Brillouin zones are sampled by a Gamma-centered Monkhorst-Pack k-point mesh with 4 × 2 × 1 for Cu(100), Cu(110), Cu(211), Cu(310), and other step surfaces constructed based on Cu(100). Brillouin zones are sampled by Gamma-centered Monkhorst-Pack k-point meshes with 3 × 4 × 1 for Cu(111), 2 × 3 × 1 for Cu(311) and Cu(322), 3 × 2 × 1 for Cu(321) and Cu(433).

For evaluating the n-C_3H_7OH selectivity of catalysts, the n-C_3H_7OH relative selectivity is defined as $K_{C2+CO}/K_{C2+H} \times K_{C3+H}$, where $K = k_{catal} / k_{(100)}$, k refers to the rate constant of an elementary reaction, "catal" refers to the catalysts, (100) refers to the perfect Cu(100) facet, "C_2+CO", "C_2+H", and "C_3+H" represent the coupling of CH_3CHO^* and CO^* to CH_3COCHO^*, the hydrogenation of CH_3CHO^* to $CH_3CH_2O^*$, and the hydrogenation of CH_3COCHO^* to $CH_3COCHOH^*$, respectively. According to the Arrhenius equation ($k = Ae^{\frac{-E_a}{RT}}$), $K = \frac{k_1}{k_2} = e^{(E_{a2}-E_{a1})/RT}$, where A, R, T, E_a refer to the Arrhenius constant, gas constant (8.314 J mol^{-1} K^{-1}), temperature (here taking the value of 298.15 K), and active energy barrier, respectively. The E_a values for those elementary steps (like "C_2+CO", "C_2+H", and "C_3+H") are estimated based on the Brønsted–Evans–Polanyi (BEP) relation.[4] Therefore, $E_a = \alpha\Delta E + \beta$, where ΔE represents the reaction energy change, α taking 0.69 for the hydrogenation steps and 0.84 for the coupling step.[5,6]

Constant potential computation

Constant potential computations are performed to analyze the influences of potential and pH conditions on the selectivity mechanism. When conducting constant potential computations, the implicit solvent environment is presented by the VASPsol code.[7,8] The relative permittivity is set to 80 to model the aqueous electrolyte. The effective surface tension parameter uses 0 to neglect the cavitation energy contribution. The linearized Poisson−Boltzmann model with a Debye length of 3.0 Å mimics the compensating charge. To consider the effect of electrode potentials during electrochemical reactions, the excess charge is added to the unit cell, and the value (Δn) is varied from −2.0 e to +2.0 e in steps of 0.5 e for all structures. Then the energy (E) of the system is related to the potential, and the relationship between E and Δn follows Equation 1. The electrode potential (U_q) is referenced to the standard hydrogen electrode (SHE) according to Equation 2. Finally, E−U_q follows a quadratic function relationship (Equation 3).[9]

$$(1)\ E = E_{DFT} - \Delta n(V_{sol} + \Phi_q/e)$$

$$(2)\ U_q(V_{SHE}) = -4.6\ V - \Phi_q/e$$

$$(3)\ E = -1/2C(U_q - U_0)^2 + E_0$$

In Equation 1, E_{DFT} is the energy calculated by VASP; another term is the energy correction of the charged system, where V_{sol} refers to the electrostatic potential of the bulk solution, and $-\Phi_q$ represents the work function. In Equation 2, 4.6 V is the work function of the H_2/H^+ couple at

standard conditions. In Equation 3, C, U_0, and E_0 are the capacitance, the potential of zero charge (PZC), and the corresponding energy of the system at PZC, respectively.

The free energy changes (ΔG) of elementary steps are calculated as $\Delta G = \Delta E + \Delta E_{ZPE} - T\Delta S$, where ΔE, ΔE_{ZPE}, T, and ΔS are the reaction energy, zero-point energies correction, temperature, and entropy change, respectively. The free energy correction of adsorbed intermediates is calculated based on the VASPKIT tool at 298.15 K.[10] ΔE is calculated by the double reference method, which is potential dependent. The chemical potential of the solvated proton and electron pair is equal to $1/2G_{H2} + eU - 0.059pH$.

Supplementary Figures and Tables

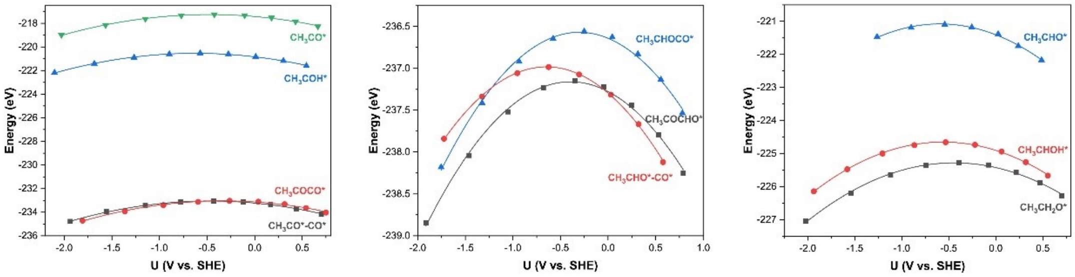

Figure S1. The fitted Energy (eV) – U (V vs. SHE) parabolic relationships of intermediates adsorbed on Cu(100).

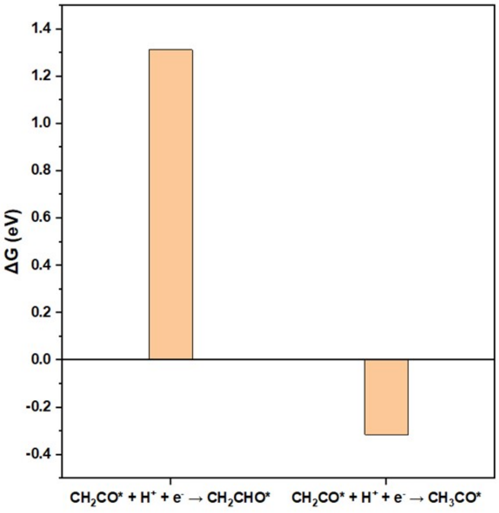

Figure S2. The free energy changes for the two possible hydrogenation steps of CH_2CO* on Cu(100).

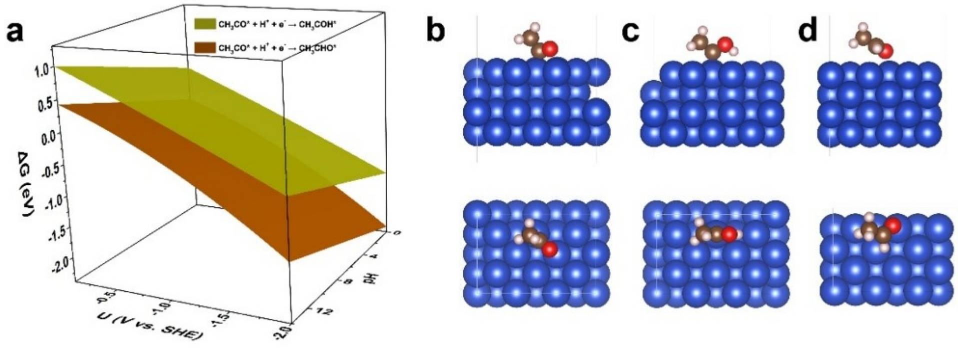

Figure S3. (a) The free energy changes of the two possible hydrogenation steps of CH_3CO^* on Cu(100) against the potential and pH. (b) The side and top views of CH_3CO^*. (c) The side and top views of CH_3COH^*. (d) The side and top views of CH_3CHO^*. Color representation in structure schematics: blue, Cu atom; brown, C atom; red, O atom; white, H atom, the below structure schematics have the same color representation.

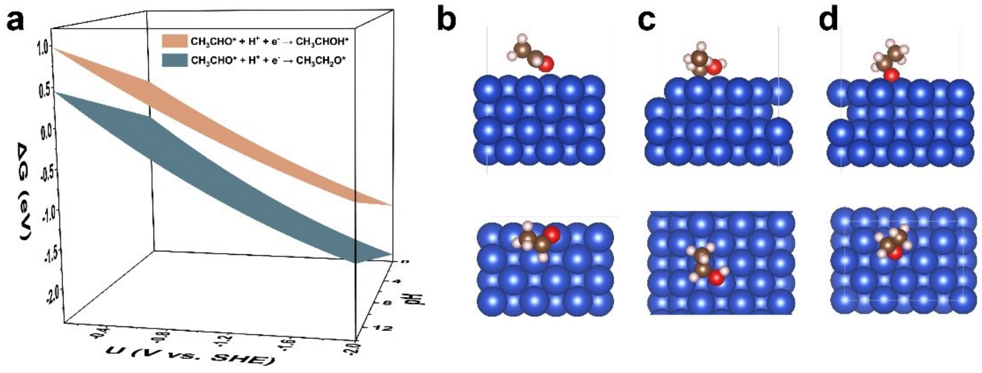

Figure S4. (a) The free energy changes of the two possible hydrogenation steps of CH_3CHO^* on Cu(100) against the potential and pH. (b) The side and top views of CH_3CHO^*. (c) The side and top views of CH_3CHOH^*. (d) The side and top views of $CH_3CH_2O^*$.

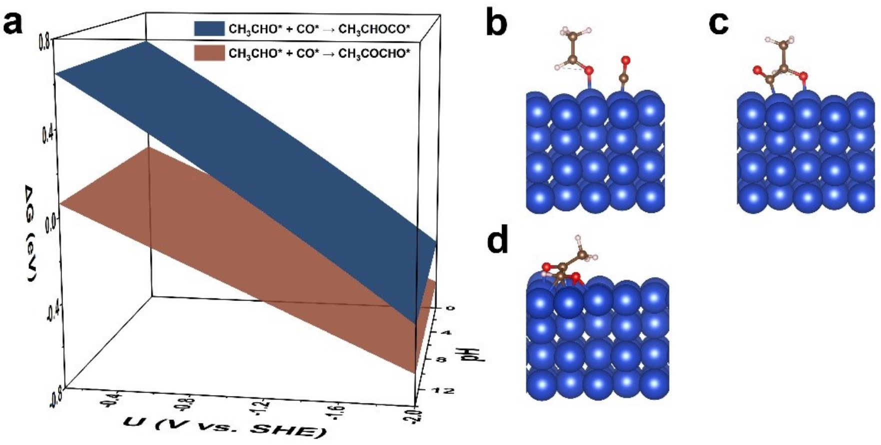

Figure S5. (a) The free energy changes of the two possible coupling steps of CH_3CHO^* on Cu(100) against the potential and pH. (b) The side view of CH_3CHO^*-CO^*. (c) The side view of CH_3CHOCO^*. (d) The side view of CH_3COCHO^*.

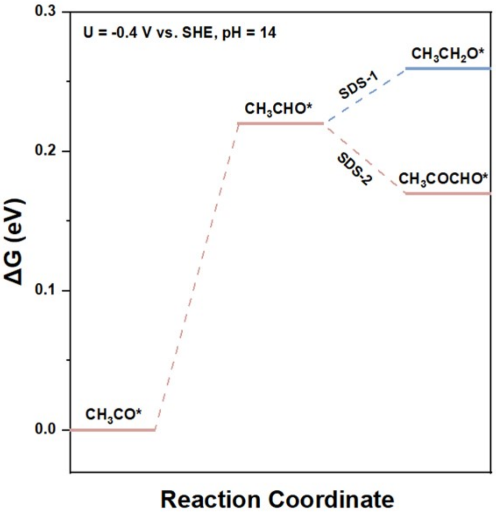

Figure S6. The free energy profiles from CH_3CO^* to $CH_3CH_2O^*$ and CH_3COCHO^* pathways, the selective-determining steps (SDSs) for the ethanol and n-propanol pathways were marked.

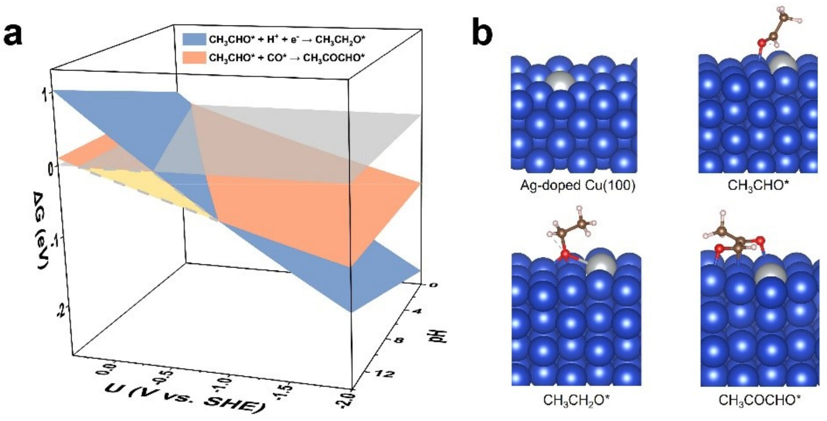

Figure S7. (a) The free energy changes of the SDSs for C_2H_5OH and n-C_3H_7OH pathways on Ag-doped Cu against the potential and pH. The grey plane is the plane with the function of $\Delta G = 0$ (eV). The region marked with the yellow color is where the coupling step dominates. (b) The side views of Ag-doped Cu and intermediates adsorbed on Ag-doped Cu, these intermediates are involved in the SDSs.

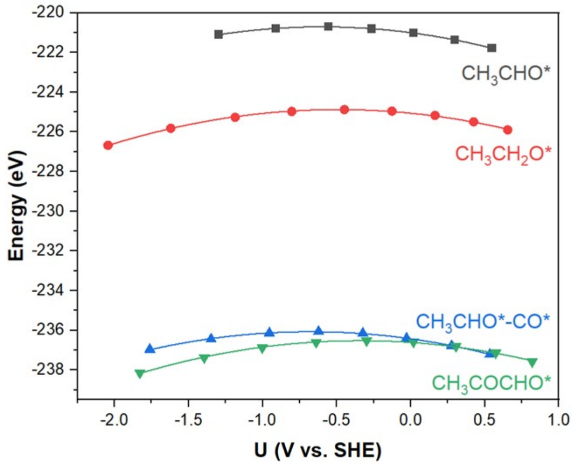

Figure S8. The fitted Energy (eV) – U (V vs. SHE) parabolic relationships of intermediates adsorbed on Ag-doped Cu.

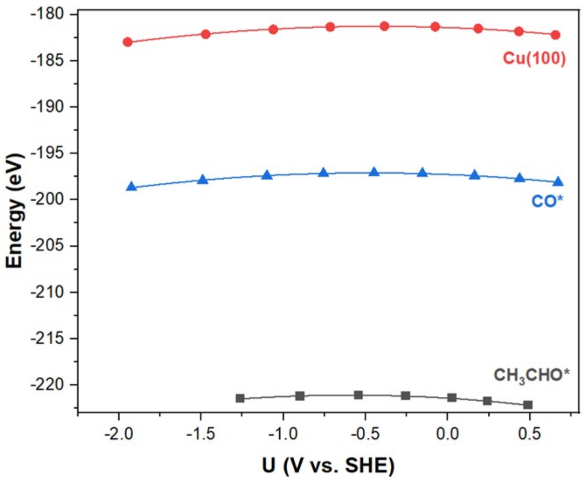

Figure S9. The fitted parabolic Energy(eV) – U (V vs. SHE) parabolic relationships of Cu(100) and intermediates adsorbed on Cu(100).

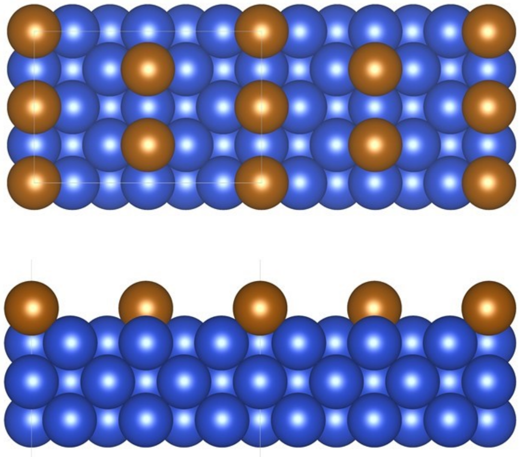

Figure S10. The top and side views of Step_u1d2.

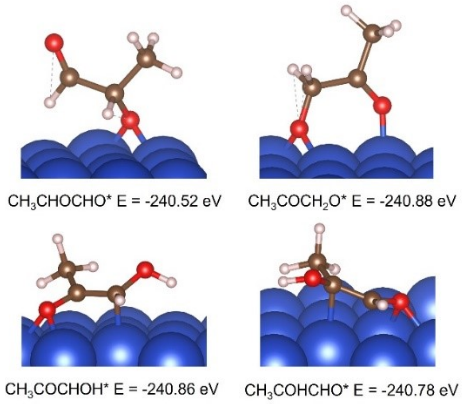

Figure S11. The possible structures formed by the hydrogenation of CH_3COCHO^* on Cu(100) and their energies. The energies of $CH_3COCH_2O^*$ and $CH_3COCHOH^*$ are very close. The methyl group of $CH_3COCHOH^*$ is closer to the surface, the intermediate may be stabilized on the step surfaces since there is more room for the methyl group to get close to the surface. Therefore, $CH_3COCHOH^*$ is chosen to be the hydrogenated structure of $^*CHOCOCH_3$.

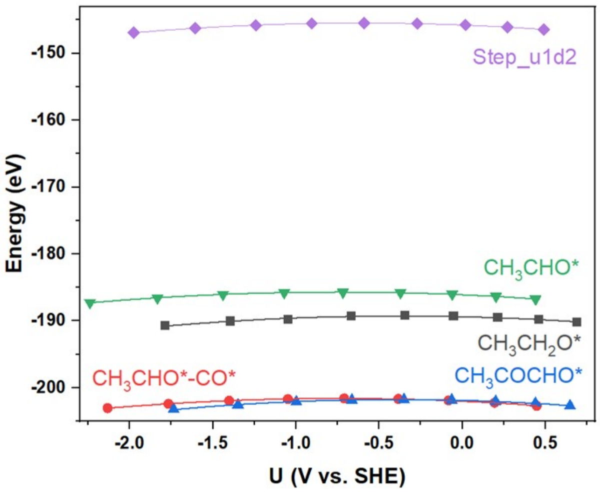

Figure S12. The fitted Energy (eV) – U (V vs. SHE) parabolic relationships of Step_u1d2, and intermediated adsorbed on Step_u1d2.

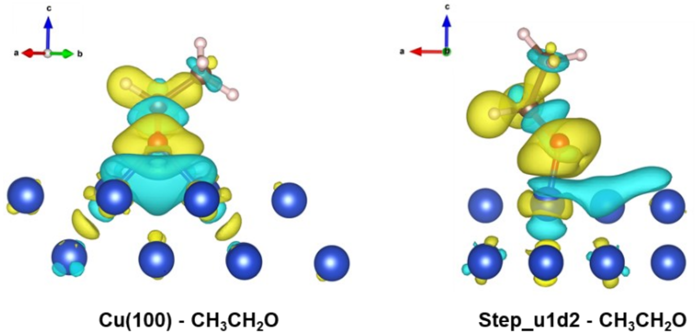

Figure S13. The differential charge density diagrams of CH_3CH_2O* on Cu(100) and Step_u1d2, where the yellow region means the accumulation of charge density and the cyan region means the attenuation of charge density. The differential charge density ($\Delta\rho$) is defined as $\Delta\rho = \rho(AB) - \rho(A) - \rho(B)$.

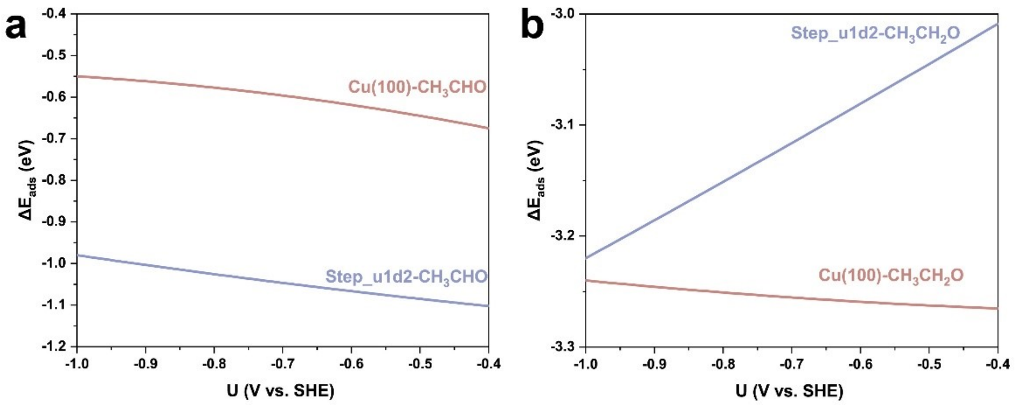

Figure S14. (a) The adsorption energies of CH_3CH_2O* on Cu(100) and Step_u1d2 versus the potential. (b) The adsorption energies of CH_3CHO* on Cu(100) and Step_u1d2 versus the potential. The potential range (–1.0 ~ –0.4 V vs. SHE) is the preferential potential range for forming n-propanol on Step_u1d2.

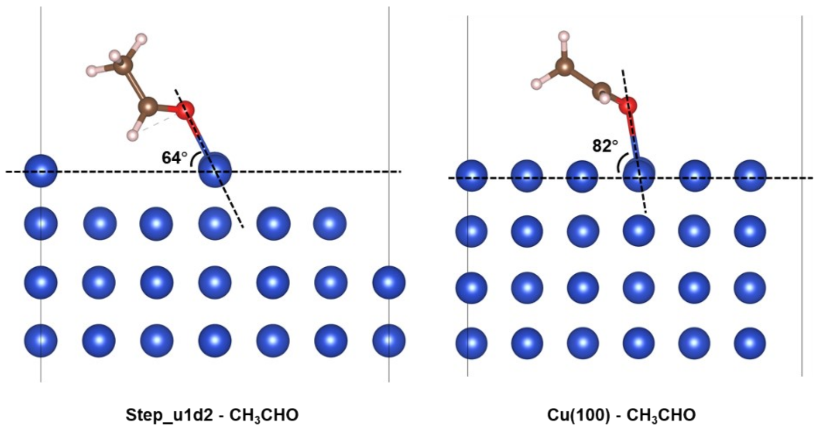

Figure S15. The adsorption configurations of CH_3CHO* on Step_u1d2 and Cu(100). The angles between the Cu-O bond and the surface plane were marked.

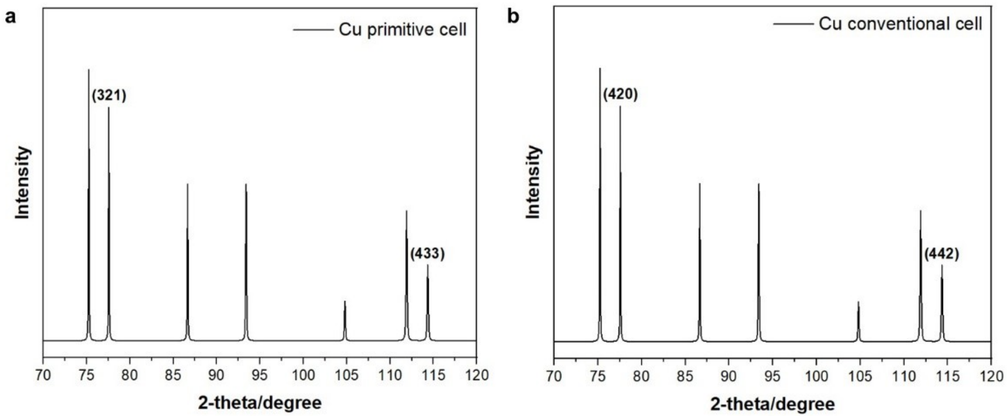

Figure S16. (a) The XRD simulation curve of Cu primitive cell using the synchrotron radiation as the XRD radiation source. (b) The XRD simulation curve of Cu conventional cell using the synchrotron radiation as the XRD radiation source.

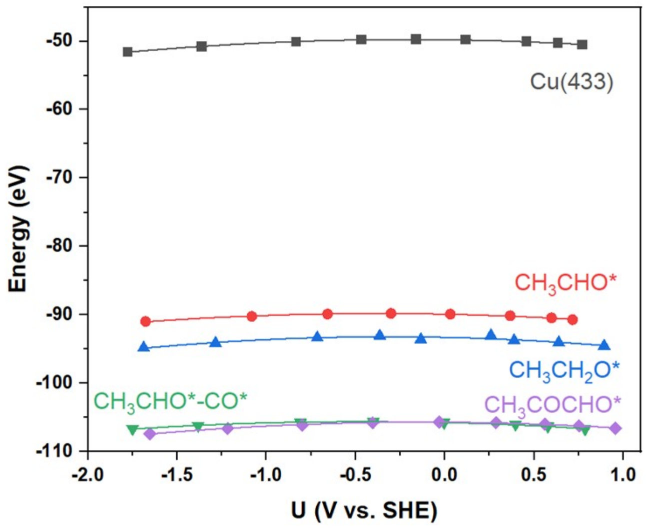

Figure S17. The fitted Energy (eV) – U (V vs. SHE) parabolic relationships of Cu(433) and intermediates adsorbed on Cu(433).

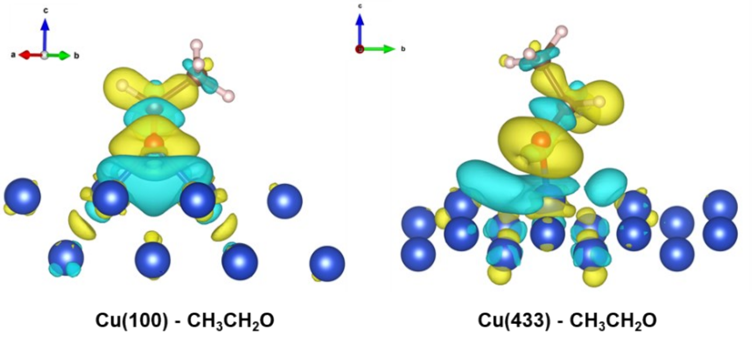

Figure S18. The differential charge density diagram of CH_3CH_2O* on Cu(100) and Cu(433), where the yellow region means the accumulation of charge density and the cyan region means the attenuation of charge density. The differential charge density ($\Delta\rho$) is defined as $\Delta\rho = \rho(AB) - \rho(A) - \rho(B)$.

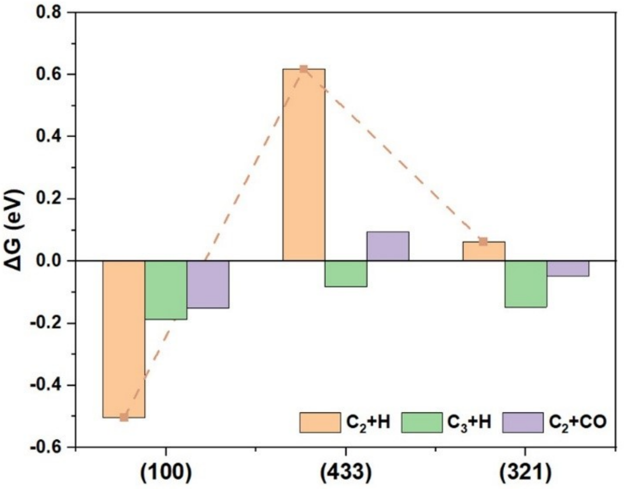

Figure S19. The free energy changes for the hydrogenation of CH_3CHO* to CH_3CH_2O*, the coupling of CH_3CHO* with CO* to CH_3COCHO*, and the hydrogenation of CH_3COCHO* to $CH_3COCHOH$*, on Cu(100), Cu(433) and Cu(321) surfaces.

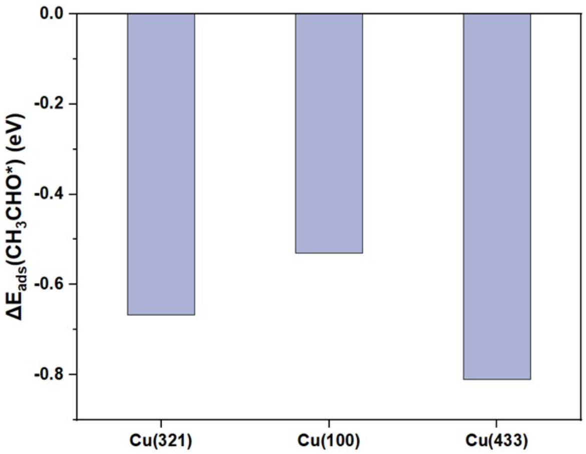

Figure S20. The adsorption energies of CH_3CHO* on Cu(321), Cu(100), and Cu(433).

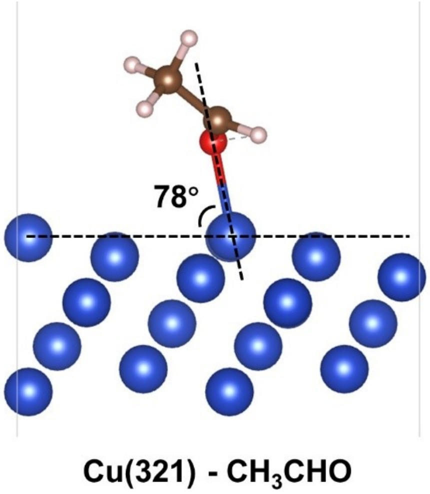

Figure S21. The adsorption configuration of CH_3CHO^* on Cu(321), the angle between the Cu−O bond and the surface plane was marked.

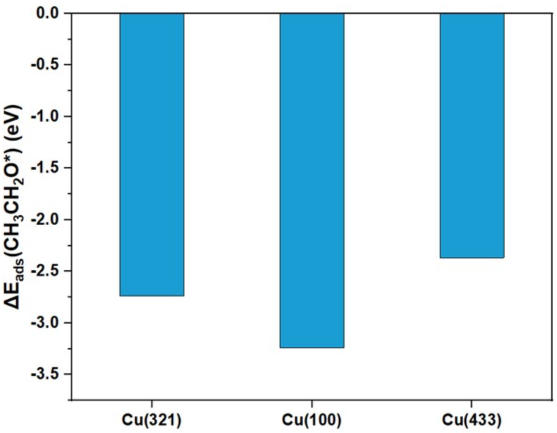

Figure S22. The adsorption energies of $CH_3CH_2O^*$ on Cu(321), Cu(100), and Cu(433).

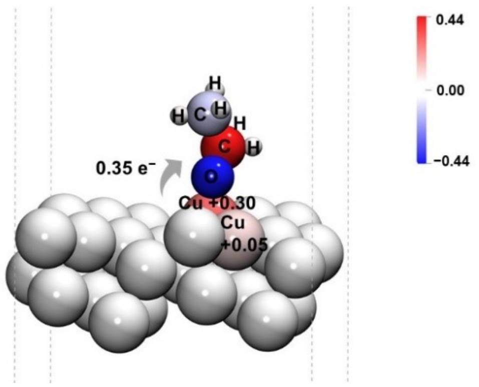

Figure S23. The atomic charge coloring diagram of $CH_3CH_2O^*$ on Cu(321).

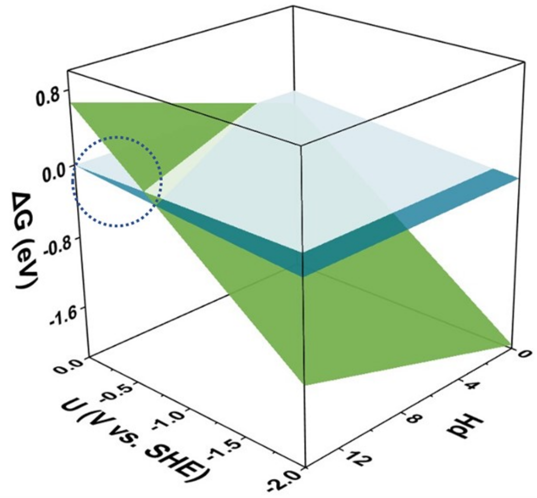

Figure S24. The free energy changes of the hydrogenation (green surface) and coupling (blue surface) steps of CH_3CHO^* on Cu(321) against the potential and pH. The dashed circle highlights the dominant potential range (at pH = 14) where the coupling step proceeds preferably. The light grey plane is the plane with the function of ΔG = 0 (eV).

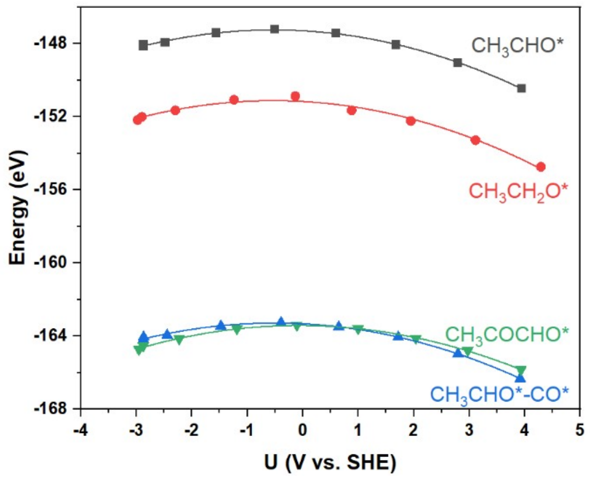

Figure S25. The fitted Energy (eV) – U (V vs. SHE) parabolic relationships of intermediates adsorbed on Cu(321).

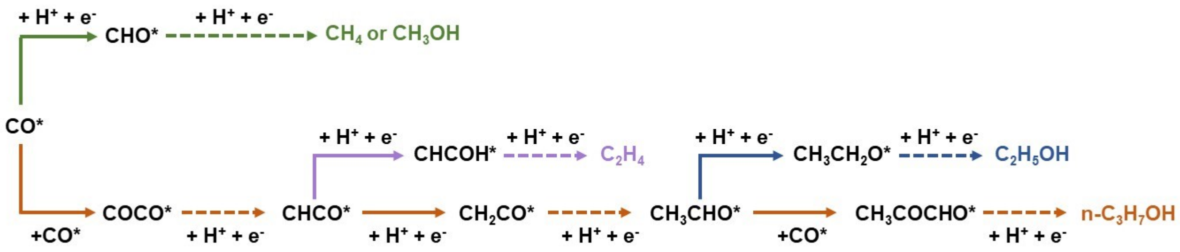

Figure S26. The reaction pathways to form methane, methanol, ethylene,[11] ethanol, and n-propanol, starting from the CO* intermediate, the dehydration processes involved in these reaction pathways were omitted. The selectivity-determining steps of these competitive products in $CO_{(2)}RR$ were highlighted with solid arrows. The n-propanol whole selectivity was calculated by considering all selectivity-determining steps in this reaction network.

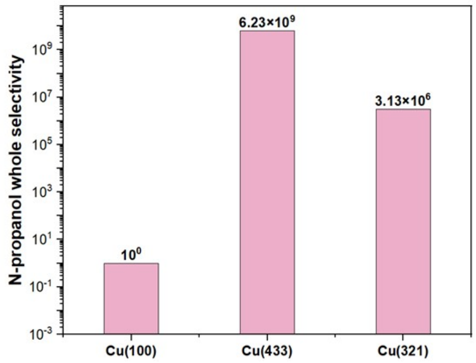

Figure S27. The whole selectivities of Cu(100), Cu(433), and Cu(321) surfaces for $CO_{(2)}$RR to n-propanol by considering the competition of methane, methanol, ethylene, and ethanol.

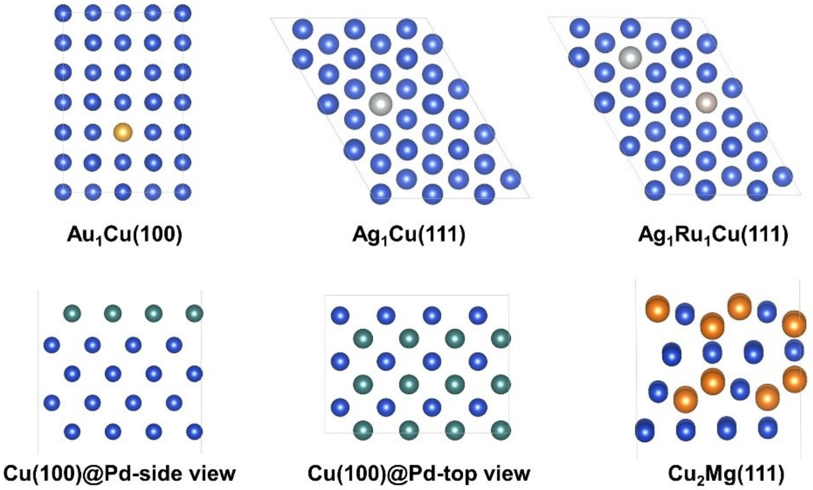

Figure S28. The structures of Cu-based bimetals shown in Fig. 4h, among which Au_1Cu(100),[12] Ag_1Cu(111),[13] and Ag_1Ru_1Cu(111)[14] are experimentally reported catalysts for $CO_{(2)}$RR to n-propanol. Cu_2Mg(111) is an experimentally reported catalyst for CO_2RR to ethanol.

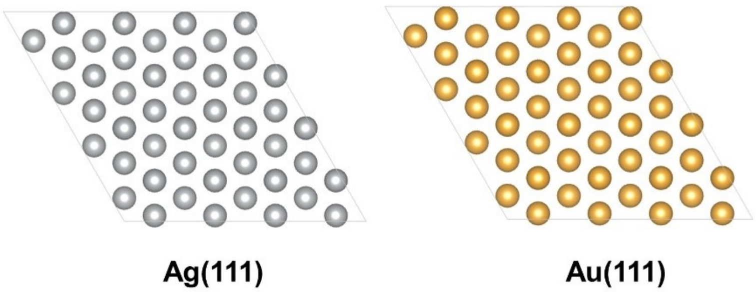

Figure S29. The structures of non-Cu metals shown in Fig. 4h.

Table S1. The Gibbs free energies of H_2(g) and H_2O(l). The DFT energies (E_{DFT}) of H_2 and H_2O are calculated in a 10 Å × 10 Å × 10 Å unit cell in vacuum.

	Pressure (atm)	Temperature (K)	E_{DFT} (eV)	ΔG (eV)	G (eV)
H_2 (g)	1	298.15	-6.759	-0.047	-6.806
H_2O (l)	0.035	298.15	-14.219	-0.002	-14.221

Table S2. The energy E (eV) – potential U (V vs. SHE) relationships of intermediates adsorbed on Cu(100).

	E – U relationship	R^2
CH_3CO*	$E = -0.73U^2 - 0.69U - 217.43$	0.996
CH_3CHO*	$E = -0.93U^2 - 1.11U - 221.40$	0.995
CH_3COH*	$E = -0.76U^2 - 0.94U - 220.83$	0.998
CH_3CO*-CO*	$E = -0.79U^2 - 0.74U - 233.23$	0.998
CH_3COCO*	$E = -0.79U^2 - 0.52U - 233.13$	0.994
CH_3CH_2O*	$E = -0.72U^2 - 0.64U - 225.42$	0.996
CH_3CHOH*	$E = -0.80U^2 - 0.89U - 224.90$	0.999
CH_3CHO*-CO*	$E = -0.75U^2 - 0.97U - 237.29$	0.999
CH_3COCHO*	$E = -0.75U^2 - 0.59U - 237.28$	0.997
CH_3CHOCO*	$E = -0.78U^2 - 0.47U - 236.64$	0.996

Table S3. The E (eV) – U (V vs. SHE) relationships of intermediates adsorbed on Ag-doped Cu.

	E – U relationship	R^2
CH_3CHO*	$E = -0.82U^2 - 0.98U - 221.00$	0.999
CH_3CH_2O*	$E = -0.75U^2 - 0.74U - 225.06$	0.998
CH_3CHO*-CO*	$E = -0.77U^2 - 1.05U - 236.43$	0.999
CH_3COCHO*	$E = -0.75U^2 - 0.52U - 236.61$	0.997

Table S4. The E (eV) – U (V vs. SHE) relationships of Cu(100), CO* and CH_3CHO* adsorbed on Cu(100), and the energies (eV) of CO and CH_3CHO molecules in a vacuum.

	E – U relationship / Energy	R^2
Cu(100)	$E = -0.75U^2 - 0.64U - 181.41$	0.996
CH_3CHO*	$E = -0.93U^2 - 1.11U - 221.40$	0.995
CO*	$E = -0.77U^2 - 0.73U - 197.27$	0.999
CH_3CHO	-39.16	-
CO	-14.80	-

Table S5. The structure information and adsorption energy data of Cu(100) and step surfaces Cu(100) constructed based on Cu(100). The unit of width and adsorption energy are Å and eV, respectively.

	The width of the upper terrace	Width of the lower terrace	$\Delta E_{ads}(CO^*)$	$\Delta E_{ads}(CH_3CHO^*)$	$\Delta E_{ads}(CH_3CH_2O^*)$	$\Delta E_{ads}(CH_3CHO^*)/\Delta E_{ads}(CO^*)$	$\Delta E_{ads}(CH_3CH_2O^*)/\Delta E_{ads}(CO^*)$
Cu(100)	12.6	0	–0.998	–0.530	–3.241	0.531	3.247
Step_u5d1	9	1.8	–1.120	–0.829	–3.690	0.740	3.295
Step_u4d2	7.2	3.6	–1.128	–0.942	–3.364	0.835	2.982
Step_u3d3	5.4	5.4	–1.095	–0.848	–3.249	0.774	2.967
Step_u2d4	3.6	7.2	–1.140	–0.781	–3.303	0.685	2.897
Step_u1d5	1.8	9	–1.151	–0.788	–2.825	0.685	2.454

Table S6. The E (eV) – U (V vs. SHE) relationships of Step_u1d2 and intermediates adsorbed on Step_u1d2, and the energies (eV) of CH_3CH_2O and CH_3CHO molecules in vacuum.

	E – U relationship / Energy	R^2
Step_u1d2	$E = -0.78U^2 - 0.96U - 145.77$	0.998
CH_3CHO^*	$E = -0.72U^2 - 1.08U - 186.09$	0.998
$CH_3CH_2O^*$	$E = -0.76U^2 - 0.58U - 189.37$	0.973
CH_3CHO^*-CO^*	$E = -0.76\ U^2 - 1.13U - 201.99$	0.999
CH_3COCHO^*	$E = -0.84U^2 - 0.66U - 201.87$	0.997
CH_3CH_2O	–40.74	-
CH_3CHO	–39.16	-

Table S7. The E (eV) – U (V vs. SHE) relationships of Cu(433) and intermediates adsorbed on Cu(433).

	E – U relationship	R^2
Cu(433)	$E = -0.75U^2 - 0.31U - 49.78$	0.996
$CH_3CH_2O^*$	$E = -0.89U^2 - 0.56U - 93.28$	0.875
CH_3CHO^*	$E = -0.71U^2 - 0.53U - 89.90$	0.990
CH_3CHO^*-CO^*	$E = -0.66U^2 - 0.61U - 105.74$	0.988
CH_3COCHO^*	$E = -0.74U^2 - 0.17U - 105.68$	0.994

Table S8. The E (eV) – U (V vs. SHE) relationships of intermediates adsorbed on Cu(321).

	E – U relationship	R^2
CH_3CH_2O*	$E = -0.16U^2 - 0.17U - 151.17$	0.984
CH_3CHO*	$E = -0.16U^2 - 0.17U - 147.30$	0.998
CH_3CHO*-CO*	$E = -0.16U^2 - 0.16U - 163.32$	0.997
CH_3COCHO*	$E = -0.15U^2 - 0.02U - 163.42$	0.998

References

1 G. Kresse and J. Furthmüller, Comput. Mater. Sci., 1996, 6, 15–50.

2 J. P. Perdew, K. Burke and M. Ernzerhof, Phys. Rev. Lett., 1996, 77, 3865–3868.

3 S. Grimme, J. Antony, S. Ehrlich and H. Krieg, J. Chem. Phys., 2010, 132, 154104.

4 J. K. Nørskov, T. Bligaard, B. Hvolbæk, F. Abild-Pedersen, I. Chorkendorff and C. H. Christensen, Chem. Soc. Rev., 2008, 37, 2163–2171.

5 S. G. Wang, B. Temel, J. A. Shen, G. Jones, L. C. Grabow, F. Studt, T. Bligaard, F. Abild-Pedersen, C. H. Christensen and J. K. Norskov, Catal. Lett., 2011, 141, 370–373.

6 S. Wang, V. Petzold, V. Tripkovic, J. Kleis, J. G. Howalt, E. Skúlason, E. M. Fernández, B. Hvolbæk, G. Jones, A. Toftelund, H. Falsig, M. Björketun, F. Studt, F. Abild-Pedersen, J. Rossmeisl, J. K. Nørskov and T. Bligaard, Phys. Chem. Chem. Phys., 2011, 13, 20760–20765.

7 K. Mathew, V. S. C. Kolluru, S. Mula, S. N. Steinmann and R. G. Hennig, J. Chem. Phys., 2019, 151, 234101.

8 K. Mathew, R. Sundararaman, K. Letchworth-Weaver, T. A. Arias and R. G. Hennig, J. Chem. Phys., 2014, 140, 084106.

9 Z. Duan and G. Henkelman, ACS Catal., 2019, 9, 5567–5573.

10 V. Wang, N. Xu, J.-C. Liu, G. Tang and W.-T. Geng, Comput. Phys. Commun., 2021, 267, 108033.

11 C. Zhan, F. Dattila, C. Rettenmaier, A. Herzog, M. Herran, T. Wagner, F. Scholten, A. Bergmann, N. López and B. Roldan Cuenya, Nat. Energy, 2024, 9, 1485–1496.

12 S. Jeong, C. Huang, Z. Levell, R. X. Skalla, W. Hong, N. J. Escorcia, Y. Losovyj, B. Zhu, A. N. Butrum-Griffith, Y. Liu, C. W. Li, D. Reifsnyder Hickey, Y. Liu and X. Ye, J. Am. Chem. Soc., 2024, 146, 4508–4520.

13 X. Wang, Z. Wang, T.-T. Zhuang, C.-T. Dinh, J. Li, D.-H. Nam, F. Li, C.-W. Huang, C.-S. Tan, Z. Chen, M. Chi, C. M. Gabardo, A. Seifitokaldani, P. Todorović, A. Proppe, Y. Pang, A. R. Kirmani, Y. Wang, A. H. Ip, L. J. Richter, B. Scheffel, A. Xu, S.-C. Lo, S. O. Kelley, D. Sinton and E. H. Sargent, Nat. Commun., 2019, 10, 5186.

14 X. Wang, P. Ou, A. Ozden, S.-F. Hung, J. Tam, C. M. Gabardo, J. Y. Howe, J. Sisler, K. Bertens, F. P. García de Arquer, R. K. Miao, C. P. O’Brien, Z. Wang, J. Abed, A. S. Rasouli, M. Sun, A. H. Ip, D. Sinton and E. H. Sargent, Nat. Energy, 2022, 7, 170–176.
